# A bioengineered organotypic prostate model for the study of tumor microenvironment-induced immune cell activation

**DOI:** 10.1093/intbio/zyaa020

**Published:** 2020-10-09

**Authors:** Sheena C Kerr, Molly M Morgan, Amani A Gillette, Megan K Livingston, Karina M Lugo-Cintron, Peter F Favreau, Logan Florek, Brian P Johnson, Joshua M Lang, Melissa C Skala, David J Beebe

**Affiliations:** Department of Pathology and Laboratory Medicine, University of Wisconsin-Madison, Madison, WI, USA; Carbone Cancer Center, University of Wisconsin-Madison, Madison, WI, USA; Department of Pathology and Laboratory Medicine, University of Wisconsin-Madison, Madison, WI, USA; Department of Biomedical Engineering, University of Wisconsin-Madison, Madison, WI, USA; Department of Chemistry, University of Wisconsin-Madison, Madison, WI, USA; Department of Pathology and Laboratory Medicine, University of Wisconsin-Madison, Madison, WI, USA; Carbone Cancer Center, University of Wisconsin-Madison, Madison, WI, USA; Department of Biomedical Engineering, University of Wisconsin-Madison, Madison, WI, USA; Morgridge Institute for Research, Madison, WI, USA; Morgridge Institute for Research, Madison, WI, USA; Department of Biomedical Engineering, University of Wisconsin-Madison, Madison, WI, USA; Carbone Cancer Center, University of Wisconsin-Madison, Madison, WI, USA; Department of Medicine, University of Wisconsin-Madison, Madison, WI, USA; Carbone Cancer Center, University of Wisconsin-Madison, Madison, WI, USA; Department of Biomedical Engineering, University of Wisconsin-Madison, Madison, WI, USA; Morgridge Institute for Research, Madison, WI, USA; Department of Pathology and Laboratory Medicine, University of Wisconsin-Madison, Madison, WI, USA; Carbone Cancer Center, University of Wisconsin-Madison, Madison, WI, USA; Department of Biomedical Engineering, University of Wisconsin-Madison, Madison, WI, USA

## Abstract

The prostate tumor microenvironment (TME) is strongly immunosuppressive; it is largely driven by alteration in cell phenotypes (i.e. tumor-associated macrophages and exhausted cytotoxic T cells) that result in pro-tumorigenic conditions and tumor growth. A greater understanding into how these altered immune cell phenotypes are developed and could potentially be reversed would provide important insights into improved treatment efficacy for prostate cancer. Here, we report a microfluidic model of the prostate TME that mimics prostate ducts across various stages of prostate cancer progression, with associated stroma and immune cells. Using this platform, we exposed immune cells to a benign prostate TME or a metastatic prostate TME and investigated their metabolism, gene and cytokine expression. Immune cells exposed to the metastatic TME showed metabolic differences with a higher redox ratio indicating a switch to a more glycolytic metabolic profile. These cells also increased expression of pro-tumor response cytokines that have been shown to increase cell migration and angiogenesis such as Interleukin-1 (IL-1) *a* and Granulocyte-macrophage colony-stimulating factor (GM-CSF). Lastly, we observed decreased TLR, STAT signaling and TRAIL expression, suggesting that phenotypes derived from exposure to the metastatic TME could have an impaired anti-tumor response. This platform could provide a valuable tool for studying immune cell phenotypes in *in vitro* tumor microenvironments.

**Insight, Innovation, Integration**

Exposure to the tumor microenvironment (TME) results in immune cell phenotype changes that contribute to immunosuppression including development of tumor-associated macrophages (TAMs) and T-cell exhaustion. Being able to prevent or reverse these phenotypes could result in improved treatment efficacy. Human immunology can be challenging to model *in vivo* due to differences between mouse and human immune systems while *in vitro* models often lack the relevant TME components. This innovative microfluidic model of the prostate TME allows spatial and temporal control of TME components, which facilitates the study of microenvironment influence on immune cell phenotypes and immunosuppression. Using this model to study immune cell phenotype changes provides potential for designing novel therapeutics or improving the efficacy of existing immunotherapies.

## INTRODUCTION

Prostate cancer is the most commonly diagnosed malignancy in men and accounts for the second most prevalent cause of cancer-driven mortality, with an estimated 33 000 deaths projected for 2020 [1]. While low-risk localized disease can be effectively treated, almost all patients with advanced symptomatic disease will develop resistance to androgen deprivation therapy (ADT) and go on to develop castrate-resistant prostate cancer (CRPC), which is considered incurable [2] with a median survival of ~42 months [3]. Concerningly, there is a rising incidence rate of men presenting with metastatic disease, with a projected 42% increase in *de novo* metastatic disease by the year 2025 [4]. One promising treatment avenue is immune checkpoint inhibition (ICI) where key negative regulators of cytotoxic lymphocyte activity are inhibited, such as CTLA-4 and PD-1, to improve the immune response against the tumor [5]. ICI has shown promising results in solid tumors such as melanoma and renal cell carcinoma [6, 7], but efficacy in metastatic prostate cancer has been limited. Ipilimumab (anti-CTLA-4) showed no significant survival benefit over placebo in CRPC patients [8], while pembrolizumab (anti-PD-1) did show anti-tumor activity but the response rates were low [9].

Prostate tumors have often been described as immunologically ‘cold’ with a relative paucity of T-cell infiltration [10]. However, in contrast to many solid tumors where ICI response rates are increased in tumors with higher levels of tumor-infiltrating lymphocytes (TIL) [11, 12], the relationship between TIL density and ICI response in prostate cancer is unclear and potentially detrimental. Studies have reported that high levels of CD8^+^ TILs in prostate cancer stroma have been associated with higher levels of immunosuppressive receptors and poorer clinical outcomes [13, 14]. This suggests that the TIL present in the prostate cancer TME have impaired functionality and may contribute to an immunosuppressive environment. Several factors could drive this immunosuppression including increased numbers of regulatory T cells (Treg) [15, 16] and TAMs [17], which often exhibit an M2 polarized phenotype and are associated with poor clinical outcomes [18]. Corruption of stromal cells such as fibroblasts to cancer-associated phenotypes results in secretion of cytokines, such as CXCL12 and TGFβ1, which can influence immune cell migration and function [19]. This interplay between tumor cells and other cells in the TME (i.e. immune, fibroblast) is critical for driving immunosuppression and tumor growth. An improved understanding as to how immunosuppressive phenotypes develop and can be targeted or reprogrammed could greatly improve efficacy of immunotherapy treatments in prostate cancer. Thus, there is a need for improved *in vitro* models that can represent the prostate TME to allow study and manipulation of immune cells derived from human samples in a system that can include multiple components of the TME as well as structures that can mimic those found *in vivo* while providing spatial and temporal control of individual compartments.

Here, we report a microfluidic *in vitro* model of the prostate TME that mimics prostate ductal structures with associated immune cells and stromal cells. Based on the LumeNEXT platform [20], lumens are molded from hydrogel and seeded with prostate epithelial cells that are surrounded by matrix-embedded fibroblasts. Culturing cells in structures that replicate *in vivo* geometry can have a profound impact on cell phenotypes and behavior. Epithelial cells cultured in lumens demonstrated a significantly enhanced secretion of chemokines and growth factors when compared to traditional 2D or 3D cultures [21]. Further, significant differences in response to the aromatase inhibitor, anastrazole, were observed between lean and obese women in a lumen model of the breast cancer microenvironment but were not detectable in a 2D culture model [22, 23]. Depending on the source of the seeded cells, models can be made to represent normal or metastatic conditions. In this model, we opted to use BPH-1-derived Cancer Progression (BCaP) cells [24]. These prostate cancer cells model a unique progression in invasiveness and metastatic potential. A fusion of BPH-1 and rodent urogenital mesenchyme, the cells were xenografted into mice which were then subjected to hormone treatment. BCaP-NT cells were recovered from a graft from an untreated mouse and represent a non-tumorigenic cell line, whereas BCaP-M1 cells were isolated from a metastatic lymph node after 4 months of hormone treatment [24]. Fibroblasts were derived from primary tumor samples or adjacent normal tissue and pooled. The model reported here is designed to examine the crosstalk between immune cells and components of the surrounding microenvironment and can report donor cell heterogeneity. To this end, peripheral blood mononuclear cells (PBMCs) are cultured within the lumen and subject to crosstalk from epithelial and stromal cells. PBMCs cultured in metastatic models exhibit changes in metabolism and phenotype, upregulating expression of pro-tumorigenic and angiogenic cytokines and downregulating genes critical to the anti-tumor response. This platform provides a unique way to investigate the development and reprogramming of immunosuppressive phenotypes in the prostate cancer TME.

## MATERIALS AND METHODS

### Cell culture

BCaP-NT and BCaP-M1 cells were a gift from William Ricke (University of Wisconsin, Madison). BCaP cells were cultured in supplemented RPMI (ThermoFisher, Waltham, MA) (containing 10% fetal bovine serum (FBS; VWR, Radnor, PA) and 1% Penicillin/Streptomycin (ThermoFisher, Waltham, MA)). PBMCs were also cultured in supplemented RPMI media.

### Primary fibroblast isolation

All tissue samples were acquired under an approved protocol by a University of Wisconsin-Madison Institutional Review Board. Biopsy punches (4 mm) were taken from normal and tumor areas of the prostate. A thin slice was taken from each punch to determine the presence of cancer, and the remaining tissue was mechanically minced and added to digestion buffer (0.5% collagenase; 0.1% Dispase; 1% PenStrep; 500 U/ml DNase 1 (Worthington Biochemical, Lakewood, NJ)) in Hepatocyte Wash Medium (Gibco, ThermoFisher, Waltham, MA) and incubated for 4 hours at 37°C. Cells were spun down and plated in FM (ScienCell, Carlsbad, CA) media at ~80 000 cells/well in a 24 well cell culture plate. Punches were confirmed to contain Normal/Tumor tissue by a certified pathologist using hematoxylin and eosin staining. Primary fibroblasts were analyzed by qPCR for expression of collagen and fibroblast activation protein (FAP) as markers of a cancer-associated phenotype (Supplementary Fig. S1).

### PBMC isolation

Healthy blood donations were obtained from the UW Carbone Cancer Center Translational Science Biocore Biobank under an approved IRB protocol. Blood was diluted 1:2 with PBS + 2% FBS and added to a 50 ml SepMate tube (Stemcell, Vancouver, BC) containing 15 ml of Lymphoprep (Stemcell, Vancouver, BC). The gradients were centrifuged at 1200*g* for 10 minutes and the PBMCs were poured into a new 50 ml conical tube. PBMCs were washed twice with PBS + 2% FBS before being stored frozen in RPMI + 40% FBS + 10% DMSO.

### Device fabrication

Fabrication of LumeNEXT devices has been previously described [20]. Briefly standard soft lithography techniques were used to form the SU-8 masters that were used as molds to pour polydimethylsiloxane (PDMS) (Dow Corning, Auburn, MI) devices. The two device layers were aligned, ethanol bonded together and 340 μM rods formed from PDMS were inserted into the device chamber. The devices were oxygen plasma treated onto a 35-mm MatTek dish (MatTek, Ashland, MA). After plasma bonding, the middle chamber of the device was closed, facing the glass while the device ports remained open. Four devices were located on each MatTek dish. Devices were UV-sterilized for 15 minutes prior to use.

### Organotypic culture preparation

To minimize evaporation, MatTek dishes were placed inside an omnitray (ThermoFisher, Waltham, MA) lined around the outer edges with water-soaked Kimwipes. To achieve maximum hydrogel adhesion to the PDMS chamber, the devices were filled via a loading port with a 2% poly(ethyleneimine) (PEI) solution (Millipore-Sigma, St Louis, MO) in deionized (DI) water and incubated for 10 minutes at room temperature. Sacrificial water was added to the outer edge of the MatTek dish to minimize evaporation. The PEI solution was aspirated and a 0.4% glutaraldehyde (GA) solution (Millipore-Sigma, St Louis, MO) in DI water was added to the device using the loading port for further 30-minute incubation at room temperature. The GA solution was aspirated, and the devices were washed extensively with DI water to remove any excess GA. A collagen solution was prepared on ice using high-density rat-tail collagen type 1 (Corning, New York, NY), which was diluted to a concentration of 6 mg/ml using 10× PBS and supplemented RPMI and neutralized with 0.5 M NaOH to a pH of 7.2. Just prior to addition, the collagen solution was diluted 1:4 to a final concentration of 4.5 mg/ml with either supplemented RPMI or prostate fibroblasts at 2500 cells/μl (final concentration 650 cells/μl). The collagen solution was added to the loading port until the central chamber was filled, taking care not to overfill collagen into the side channels. Collagen was polymerized at room temperature for 10 minutes, and supplemented RPMI was added to the two side channels. The devices were incubated at 37°C for at least 1 hour to allow collagen to fully polymerize. After this incubation, a drop of supplemented RPMI was added to the input port at the bottom of the device and the PDMS rod was removed with tweezers to leave a molded lumen that connected the input and output ports. 2 μl of a 50 000 cell/μl BCaP cell suspension was added to the lumen through the input port. 5 μl of supplemented RPMI was added to each of the side channels. The devices were incubated at 37°C and rotated from top to bottom every 20 minutes for a total of four rotations to allow for cell attachment before being cultured overnight. The next morning, supplemented RPMI was added to the input port and non-adherent cells were removed from the output port. To add PBMC into organotypic culture, the cells were thawed and resuspended in supplemented RPMI at a concentration of 40 000 cells/μl. 2.5 μl of cells were added into the input port where they entered the BCaP lumen. The devices were cultured for further 4 days, changing the media twice daily through the side channels.

### Immunofluorescent staining

Cultures were fixed with 4% paraformaldehyde (Electron Microscopy Sciences, Hatfield, PA) for 15 minutes and then washed twice with PBS. To evaluate lumen morphology, cultures were stained with 33 μM Texas red phalloidin (ThermoFisher, Waltham, MA) and the nuclear stain Hoechst overnight, to stain F-Actin, and washed with PBS prior to imaging. For antibody labeling, cultures were permeabilized with 0.2% Triton X-100 for 20 minutes prior to staining. To investigate apical–basal polarity, cells were stained for the apical marker GM130 (1:50, rabbit monoclonal [clone EP892Y], Abcam, Cambridge, MA) and basal marker laminin-5 (1:50, mouse monoclonal [clone P3H9–2] Abcam, Cambridge, MA). To evaluate E-cadherin, cultures were stained with an anti E-cadherin antibody (1:50, rabbit polyclonal ab15148, Abcam, Cambridge, MA). After a 48-hour incubation with primary antibodies, cultures were washed five times over a 24-hour period with 3% BSA in PBS and then incubated with anti-rabbit Alexa-fluor-488 (1:50, ab150113, Abcam, Cambridge, MA) and/or anti-mouse Alexa-Fluor-647 (1:50, #A-21244, (ThermoFisher, Waltham, MA) for 48 hours in 3% BSA in PBS with 0.1% TWEEN-20, and all cultures were stained with the nuclear stain Hoechst. To obtain cross-sectional images of the lumens, cross-sectioning was performed as previously described [25]. Briefly, after fixation and staining, organotypic cultures were embedded in a 3% low-melting agarose solution (IBI Scientific, Dubuque, IA) and then glued to a mounting block. A VT-300 Compresstome (Precisionary Instruments, Greenville, NC) was used to cut 100-μm-thick cross-sections. The majority of images were taken with a Nikon Eclipse TI microscope (Melville, New York). To capture higher resolution images, an optimal workstation built around a Nikon Eclipse TE300 was used to perform multiphoton laser scanning microscopy as previously described [20].

### Cell proliferation assay

The CellTiter Glo assay (Promega, Madison, WI) was used to evaluate the number of viable cells in each culture following the manufacturer’s instructions. Briefly, cultures were removed from the incubator and kept at room temperature for 20 minutes prior to the assay. CellTiter Glo Reagent was made by reconstituting the CellTiter-Glo Substrate with the CellTiter-Glo Buffer. Media were aspirated from the lumens then replaced with the CellTiter Glo Reagent and incubated for 10 minutes at room temperature protected from light. Luminescence was measured with a Biorad gel imager.

### Invasiveness quantification

Brightfield images were taken of cultures every day over the course of 3 days. Cells that invaded out of the lumen and into the surrounding matrix were counted manually using Image J.

### Viability staining

To determine immune cell viability, PBMCs were recovered from the lumen and added into a 384-well plate containing a solution of 5 μM Calcein AM (ThermoFisher, Waltham, MA) and ethidium homodimer 1:200 (PromoCell, Heidelberg, Germany) in PBS. Cells were stained for 30 minutes at 37°C before imaging on a Nikon Eclipse TI microscope. To stain BCaP lumens, cultures were stained using Calcein AM and ethidium homodimer as described above adding the dye through the input port.

### Label-free redox imaging

Autofluorescence images were taken on a custom-built inverted multiphoton microscope (Bruker Fluorescence Microscopy, Middleton, WI). The system consists of a tunable laser (Chameleon Ultra II, Coherent), an inverted microscope (Nikon, Eclipse Ti) and a 40× water immersion (1.15NA, Nikon) objective. NAD(P)H and FAD images were acquired sequentially for the same field of view. An excitation wavelength of 750 nm and an emission filter of 440/80 nm were used to isolate NAD(P)H fluorescence. FAD fluorescence was isolated using an 890-nm excitation wavelength and a 550/100-nm emission bandpass filter. Fluorescence images were collected using time-correlated single-photon counting electronics (SPC-150, Becker and Hickl) and a GaAsP photomultiplier tube (H7422P-40, Hamamatsu). A pixel dwell time of 3.6 μs was used to acquire 512 × 512 pixel images over 45-second total integration time. The photon count rates were maintained at 2 × 10^5^–9 × 10^5^ photons/s to ensure adequate photon collection and no photobleaching.

### Calculation of single-cell redox ratio

The optical redox ratio was calculated from the NAD(P)H and FAD data by summing the photons detected at each pixel in the image to compute the total intensity. The intensity of NAD(P)H was then divided by the intensity of FAD for each pixel.

An automated cell segmentation pipeline was created in Cell Profiler as previously described [26]. For immune cell segmentation, a mask of the lumen was manually generated and then a customized Otsu Global threshold code identified pixels belonging to cellular regions by identifying areas within the lumen brighter than background signal. Filtering based on object sphericity was done to limit the analysis of dead cells and debris; the resulting round objects were stored as a mask. Values for the intensities of NAD(P)H and FAD as well as the redox ratio were measured for each immune cell. Normalized redox ratio was calculated by dividing each individual cell redox ratio by the average of the benign condition on the same day.

### Generation of population distributions

The collective cell population of each immune cell condition (benign, NT + NF; metastastic M1 + CAF) was input into a Gaussian mixture distribution model (MATLAB, version 2017a, MathWorks, Natick, MA) as described in [27].

$$ f\left(y;{\Phi}_g\right)=\sum \limits_{i=1}^g{\pi}_i\phi \left(y;{\mu}_i,{V}_i\right) $$

Where *g* is the number of subpopulations, ϕ(*y; μ_i_, V_i_*) is the normal probability density function with a mean of *μ_i_* and a variance *V_i_*, and *π_i_* is the mixing proportion. Goodness of fit was calculated given a set of subpopulations (*g* = 1, 2, or 3) using an Akaike information criterion [28]. The number of subpopulations was determined based on the lowest Akaike score. Finally, the probability density functions were normalized to ensure that the area under the curve for each treatment group was equal to 1.

### Qiagen RT profiler array analysis of PBMC

PBMCs were removed from the lumen via the output port and placed into an Eppendorf tube pooling the contents of four lumens. The volume was increased to 100 μl with PBS + 2 mM EDTA + 0.5% BSA (PBE). 5 μl of anti-CD45 biotin (H130, Biolegend, San Diego, CA) was added and the samples were rotated for 15 minutes at 4°C. Sera-mag speed beads streptavidin particles (GE, Marlborough, MA) were washed with PBE twice and resuspended in PBE at their original volume. 10 μl of sera-mag beads were added to the samples and incubated for another 10 minutes with rotation at 4°C. The bound cells were isolated using a magnetic pulldown, washing twice with PBE prior to lysis with RLT plus (Qiagen, Germantown, MD) + β-mercaptoethanol. RNA was isolated using the RNeasy Plus Micro Kit (Qiagen, Germantown, MD) and quantified with a pico chip on an Agilent Bioanalyzer (Santa Clara, CA). RNA integrity numbers for all samples were above 9, indicating RNA preparations of very good quality. RNA concentrations were normalized between samples for each donor, and 1–5 ng of each sample was used to make cDNA using the RT^2^ PreAMP cDNA Synthesis Kit (Qiagen, Germantown, MD) according to the manufacturer’s instructions. Samples were preamplified using the same kit with a pathway primer mix for the RT^2^ cancer inflammation and immunity crosstalk array (Qiagen, Germantown, MD). qPCR was run on the RT^2^ profiler cancer inflammation and immunity crosstalk array (Qiagen, Germantown, MD) to measure a panel of 84 different genes, according to the manufacturer’s instructions on a Roche Lightcycler 480 (Roche, Indianapolis, IN). All gene expression data were in the linear portion of the amplification curve. Samples that could not generate signal after 45 cycles were excluded from analysis. Gene expression data were compared and normalized against the geometric mean of five housekeeping genes (β-actin, β-microglobulin, GAPDH, HPRT1 and RPLP0) as previously described [29]. To normalize between donors, for each gene, the control expression was defined as 100% and the percentage expression of the experimental conditions were calculated and compared to control.

### qPCR analysis of fibroblasts

RNA was extracted from normal and tumor fibroblasts from the three patients used in the model, using an RNeasy Mini Kit (Qiagen, Germantown, MD). RNA concentration was assessed using a Nanodrop (ThermoFisher, Waltham, MA). cDNA was produced using the High Capacity RNA-to-cDNA Kit (ThermoFisher, Waltham, MA) according to manufacturer’s protocols. 25 ng was used for each qPCR reaction using primers directed against Collagen (Hs00164004_m1), FAP (Hs00990791_m1) and GAPDH (Hs00990791_m1), all purchased from ThermoFisher, Waltham, MA. qPCR was run on a Roche Lightcycler 480 (Roche, Indianapolis, IN) using Roche Lightcycler master mix according to manufacturer’s protocols. Gene expression was normalized using the delta-delta Ct method.

### Protein secretion analysis using MAGPIX

Culture media were collected from the LumeNEXT models after 4 days of culture and pooled from three devices per condition. Media were centrifuged at 300*g* for 5 minutes to remove cells. The supernatant was transferred to a microcentrifuge tube and stored at −80°C until use. Samples were thawed, diluted 1:3 with culture media and analyzed in triplicate using a custom ProcartaPlex Human Assay (ThermoFisher, Waltham, MA) following the manufacturer’s protocols. Plates were read using a Luminex MAGPIX instrument (Luminex, Austin TX). The soluble factor concentrations in media were calculated using mean fluorescence intensities (MFI) by creating a standard curve for each analyte using a five-parameter logistic curve fit. The media control background was subtracted from the net MFI for each of the wells, and each analyte was plotted against the assay standard readings, which were transformed using Log10. Nonlinear curves were fitted to the plots and the analyte concentrations were interpolated from these curves.

### Statistical analysis

All the experiments were repeated at least three times or used five biological replicates. Statistical analysis between two groups was performed using the Student’s *t*-test or using one-way analysis of variance for analysis of three groups. Statistical analyses were performed using GraphPad Prism version 7 (GraphPad Software, La Jolla, CA) and statistical significance was set at *P* < 0.05.

## RESULTS

### Development of an organotypic model of the prostate cancer microenvironment

**Figure 1:**
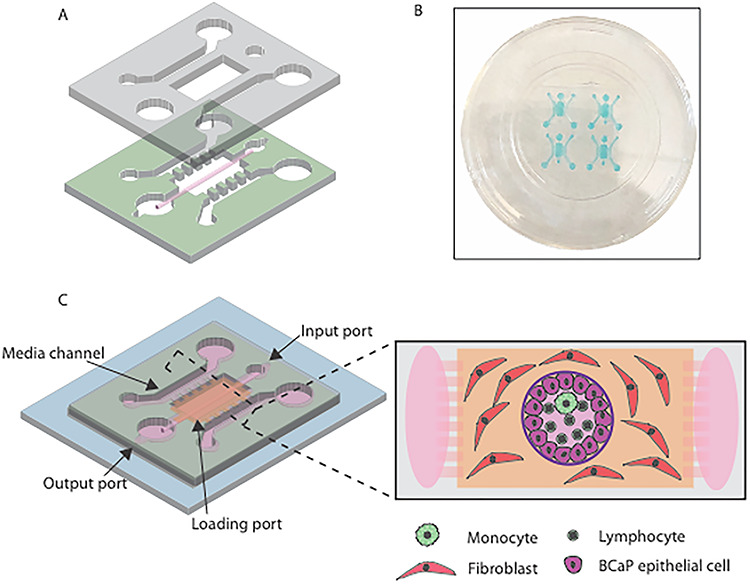
Overview of organotypic model of a prostate duct. (A) Two-layered devices were created from PDMS using the LumeNEXT method. (B) Image of a device array using colored water for visualization purposes. (C) Detailed diagram of the device showing TME model. The two PDMS layers are bonded together around a rod allowing creation of a lumen structure molded from a collagen hydrogel (orange). Lumens are lined with benign or metastatic BCaP epithelial cells (purple). PBMCs (green) are added inside the lumen. Media channels allow exchange of cell culture media (pink) from the side channels without perturbing the immune cells in the lumen.

The tumor microenvironment (TME) has a significant impact on immune cell phenotype and function. To develop a model of the prostate TME and elucidate mechanisms of immune cell dysfunction, we based the microdevice design on the LumeNEXT platform. Using this platform, hollow cylindrical structures (i.e. lumen) can be molded within a hydrogel [20, 25, 30–33], which allows recapitulation of the structure of the prostate duct. The device is constructed from two PDMS layers that are assembled around a PDMS rod (Fig. 1A). The device layers are bonded together and plasma bonded to a MatTek dish, allowing an array of four devices per dish (Fig. 1B). Injection of high-density rat-tail collagen I into the loading port, followed by removal of the rod after collagen polymerization, molded the lumen. The lumens were then seeded with prostate epithelial cells to form duct structures. To minimize the potential for variation between human donors, as our focus is on immune cell phenotype, we opted to use BCaP cells to seed the prostate ducts (Fig. 1C). BCaP cells model a unique progression in invasiveness and provide a mechanism by which to create benign or metastatic prostate cancer environments. The platform design allows incorporation of stromal cells such as fibroblasts to be embedded in the matrix surrounding the lumen (Fig. 1C). We used a pool of three matched donor primary prostate fibroblasts isolated from prostatectomy samples, using fibroblasts isolated from normal prostate tissue for the benign models and cancer-associated fibroblasts from tumor tissue for the metastatic model. A pathologist confirmed that the tissue punches used for cell isolation were normal or tumor, respectively. Fibroblasts were examined by qPCR to determine expression of collagen and FAP. While there was significant heterogeneity between the donors, donors 1 and 3 showed upregulation of collagen and donors 1 and 2 upregulated FAP (Supplementary Fig. S1), suggesting that the pooled fibroblasts contain a heterogenous mix of cells with a cancer-associated phenotype. To allow addition of immune cells inside the duct while permitting media exchange to take place without perturbation of the center of the lumen, the design of the microdevice was modified to add two media channels (Fig. 1C). These media channels are in direct soluble factor communication with the main chamber and allow media changes (or sampling) without disturbing the duct (Fig. 1). Therefore, this organotypic model system can co-culture prostate ducts, stroma and immune cells creating benign or metastatic environments.

### BCaP-M1 cells form lumens with an increased invasive phenotype

**Figure 2:**
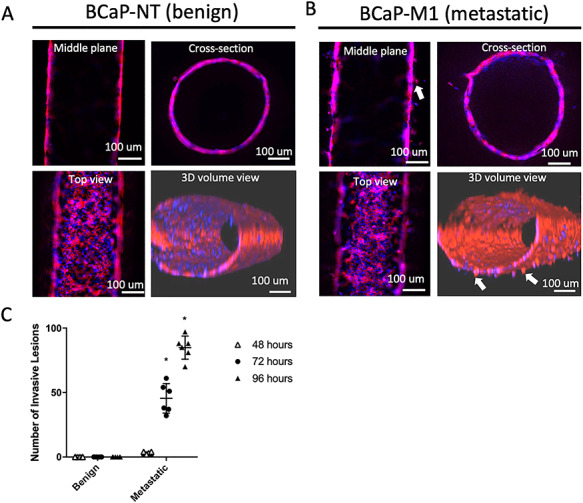
LumeNEXT and BCaP cells can create benign and metastatic prostate ductal structures. (A) LumeNext allows the molding of lumen structures from collagen hydrogel that can be lined with (A) benign or (B) metastatic BCaP prostate epithelial cell lines. Cells shown are stained with Hoechst (blue) or Phalloidin (red). White arrows highlight examples of invasive projections. (C) Quantification demonstrates that metastatic ducts have increased numbers of invasive projections. Data shown are from six independent replicates ±SD, ^*^P ≤ 0.05

Prostate ducts were formed by seeding the lumens with either the non-tumorigenic BCaP-NT or metastatic BCaP-M1 cells (Fig. 2). After a 4-day culture period, staining with phalloidin and Hoechst was performed to visualize cell morphology and showed that BCaP-NT cells form a tightly packed lumen with uniform cell organization and a hollow structure in cross-section (Fig. 2A). However, BCaP-M1 cells exhibit a more dysregulated organization with invasive projections visible on the lumen wall (Fig. 2B). Quantification of invasive lesions over a 4-day culture period by microscopy demonstrated a significant increase in lesions by 72 hours of culture that further increased by 96 hours of culture (Fig. 2C).

### BCaP-M1 cells retain their metastatic phenotype in organotypic culture

**Figure 3:**
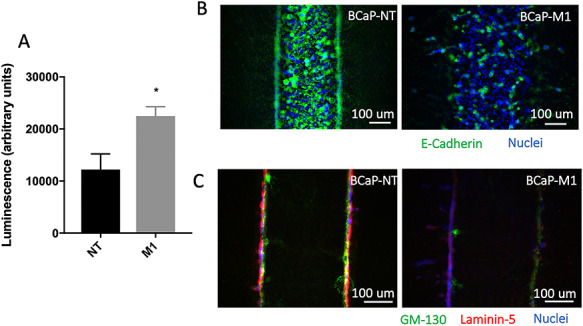
BCaP-M1 cells retain their metastatic phenotype in organotypic culture. (A) BCaP-M1 lumens (metastatic) have increased proliferation in a Cell Titre Glo proliferation assay. Bars show average ± SD, ^*^P ≤  0.05. (B) BCaP-M1 lumens stained with anti-E-Cadherin (green) and Hoechst (blue) show decreased tight junctions. (C) BCaP-M1 lumens stained with anti-GM130 (apical, green), laminin-5 (basal, red) and Hoechst (blue) show a loss of apical–basal polarity.

We next tested the rates of proliferation between the BCaP-NT and BCaP-M1 cells in 3D organotypic culture. Cell proliferation in the lumens was measured using a Cell Titer Glo assay, which demonstrated that the metastatic BCaP-M1 cells had a significantly higher rate of cell proliferation compared to the non-tumorigenic cells (Fig. 3A). A reduction in expression of the epithelial adhesion protein E-cadherin is associated with metastatic potential [34]. We stained BCaP-NT and BCaP-M1 lumens with an antibody against E-cadherin which showed reduced E-cadherin staining in the metastatic BCaP-M1 lumens (Fig. 3B). The loss of apical–basal polarity is another hallmark of cancer progression [35, 36]. We stained the benign and metastatic lumens with apical (GM-130) and basal markers (Laminin-5). BCaP-NT lumens showed a clear separation between apical and basal stains; however, the BCaP-M1 lumens showed reduced staining and mixed separation, suggesting a loss of apical–basal polarity in these cultures (Fig. 3C). Taken together, these data suggest that the BCaP-M1 cells retain their metastatic phenotype in the 3D organotypic culture.

### Inclusion of stromal cells into the organotypic model

**Figure 4:**
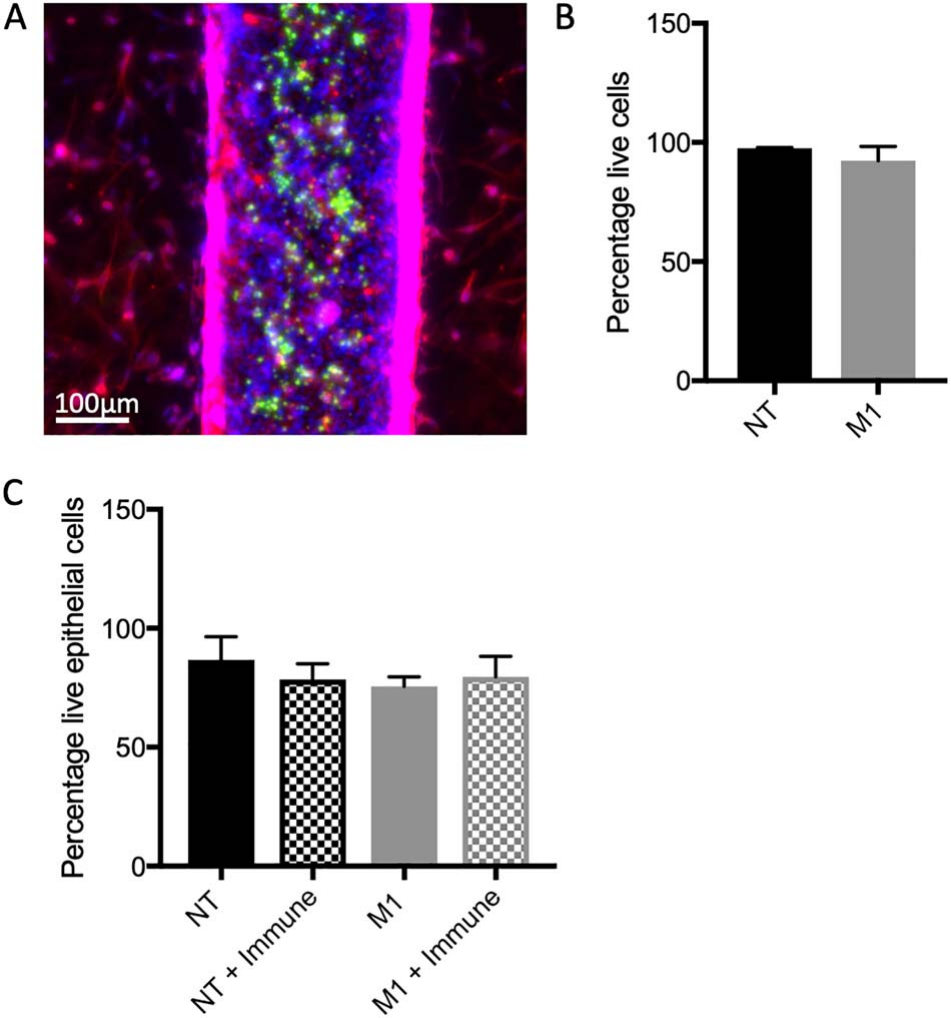
Cells retain high viability in the organotypic TME model. (A) To recreate the prostate duct TME, prostate fibroblasts are embedded in the matrix surrounding the lumen (both stained with phalloidin in red and Hoechst in blue) and immune cells (green) are added inside the lumen. (B) Immune cells cultured inside the organotypic model retain high viability. (C) There are no significant differences between viability in benign or metastatic lumens when cultured with immune cells. Bars represent average ± SD.

Benign organotypic models were created with BCaP-NT cells and normal prostate fibroblasts, while metastatic organotypic models were formed from BCaP-M1 cells and prostate cancer-associated fibroblasts. For both organotypic models, fibroblasts were embedded in the matrix surrounding the lumen where they retained their spindle-like morphology (Fig. 4A). In addition, PBMCs from normal donors were added into the center of the lumens and cultured for 4 days (Fig. 4A). Peripheral blood cells from cancer patients have been shown to have differences in some immune cell phenotypes compared to normal donors [37]; therefore, normal PBMCs were used in order to see the maximal environment-induced changes. After the 4-day culture period, the immune cells were stained with viability dyes, which showed that at least 95% of the immune cells remained viable in both culture conditions (Fig. 4B). We also investigated the viability of the epithelial cells in the lumen with and without immune cells present in the lumen and noted no significant differences, suggesting that there is little or no cytotoxic killing of the epithelial lumen by the immune cells (Fig. 4C).

### Immune cells cultured in the metastatic environment exhibit metabolic changes compared to the benign environment

**Figure 5:**
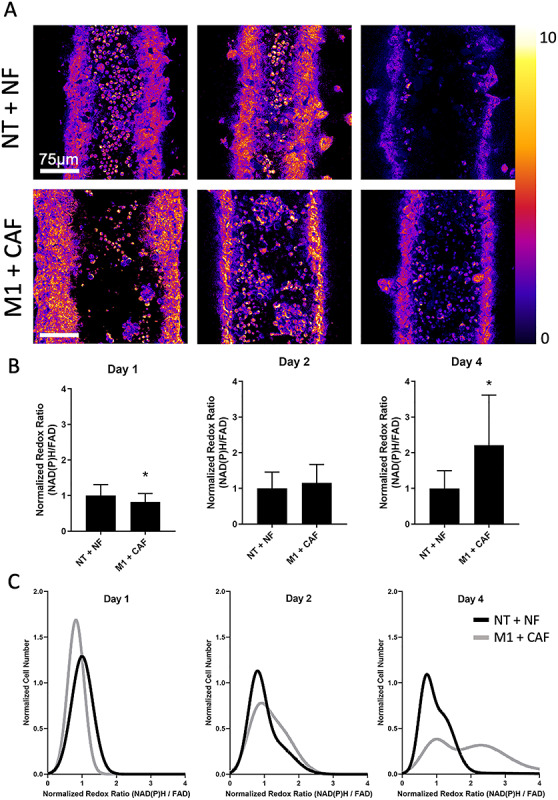
Exposure to the metastatic microenvironment causes changes to the optical redox ratio of immune cells. (A) Representative images of the optical redox ratio (intensity of NAD(P)H/intensity of FAD) of the lumens. (B) Normalized redox ratio increases in metastatic lumens over time when compared to the benign condition. (C) Sub-population analysis shows that the benign lumens shift towards a lower redox ratio than the metastatic lumens by day 4, when two distinct subpopulations of cells, including one high normalized redox ratio population, are apparent in the metastatic lumens. ^*^P < 0.005.

Immune cells in benign or metastatic models were imaged using two photon microscopy to record autofluorescence measurements of free and protein-bound NAD(P)H and FAD (Fig. 5A). PBMCs, composed predominantly of T cells and monocytes, were imaged after addition (day 1) and after 2 and 4 days of co-culture in the model (Fig. 5A). The ratio of NAD(P)H intensity to FAD intensity was then used to compute the redox ratio. The redox ratio of the PBMC in both the benign and metastatic microenvironments decreased over the culture period (Supplementary Fig. S2). However, relative changes between benign and metastatic microenvironments are apparent on days 1 and 4 when looking at the normalized redox ratio (Fig. 5B). Single-cell analysis shows that two distinct PBMC metabolic subpopulations arise in the metastatic lumens on day 4, which suggests that the different microenvironments are driving differentiation of different cell populations (Fig. 5C). Immune cells exposed to the metastatic microenvironment show a distinct, high normalized redox ratio population on day 4 compared to the benign immune cells on the same day, which is consistent with activated T cells or TAMs [38, 39].

### Immune cells exposed to the metastatic microenvironment upregulate gene expression and cytokine secretion that induces cell proliferation, migration, angiogenesis and immunosuppression

To investigate the effects of the metastatic microenvironment on immune cells, we cultured PBMC (*n* = 5 independent donors) in benign or metastatic microenvironments for 4 days. CD45^+^ cells were recovered from the lumen and RNA was extracted. Gene expression profiling was performed using the Human Cancer Inflammation and Immunity Crosstalk RT^2^ profiler array (Qiagen) to assess a panel of 84 genes and 5 housekeeping genes. Gene expression data were compared and normalized against the geometric mean of five housekeeping genes (β-actin, β-microglobulin, GAPDH, HPRT1 and RPLP0) as previously described [29]. To normalize between donors, for each gene, the control expression (benign microenvironment) was defined as 100% and the expression of the metastatic microenvironment was calculated and compared to control.

**Figure 6:**
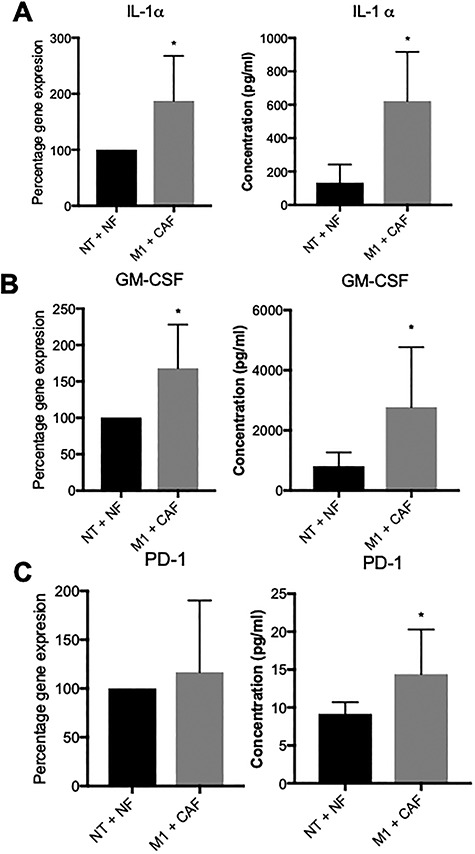
Exposure to the metastatic microenvironment causes changes to cytokine expression and impairs signaling. qPCR gene expression and MAGPIX multiplex bead-based ELISA measurements of (A) IL-1α, (B) GM-CSF and (C) PD-1. Bars represent averages ± SD, ^*^P < 0.05.

Several genes demonstrated significantly altered gene expression between immune cells exposed to the metastatic and benign microenvironments. Immune cells exposed to the metastatic microenvironment upregulated gene expression of cytokines that are associated with increased angiogenesis and tumor progression, including IL-1α (Fig. 6A), and tumor proliferation, migration and recruitment of myeloid-derived suppressor cells (MDSC) such as GM-CSF (Fig. 6B). MAGPIX multiplex bead-based ELISA profiling confirmed increased expression of these proteins secreted into the culture media (Fig. 6A and B). Additionally, gene expression of PD-1 trended but was not significantly increased; however, soluble PD-1 was significantly increased in culture supernatants (Fig. 6C and D). These data suggest that exposure to the metastatic microenvironment caused immune cell phenotypes to become altered and induced expression of pro-tumor and pro-angiogenic factors involved in the recruitment of immune suppressor cells.

### Immune cells exposed to the metastatic microenvironment show impaired toll-like receptor and STAT signaling

**Figure 7:**
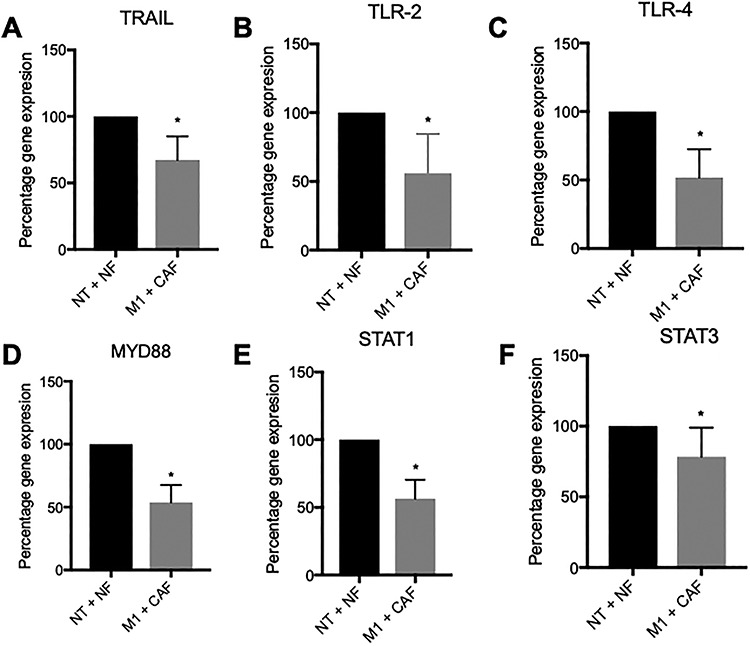
TLR and STAT signaling is impaired in immune cells exposed to the metastatic microenvironment. Gene expression of (A) TRAIL, (B) TLR2, (C) TLR4, (D) MYD88, (E) STAT1 and (F) STAT3 is downregulated. Bars represent averages ± SD, ^*^P < 0.05.

Further gene expression analysis of the CD45^+^ cells demonstrated that upon exposure to the metastatic microenvironment, TRAIL, which can induce apoptosis of tumor cells either as a secreted factor or expressed on NK and T cells, was significantly downregulated (Fig. 7A). Further, gene expression of receptors from the TLR signaling pathway were also significantly altered. Expression of TLR2 (Fig. 7B), TLR4 (Fig. 7C) and MYD88 (Fig. 7D) were all downregulated. Additional signaling pathways involved in the anti-tumor immune response were also impaired as STAT1 and STAT3 were also significantly downregulated (Fig. 7E and F).

## Discussion

In this study, we report a microfluidic model of the prostate TME that allows controlled study of human immune cell phenotypes and investigates how they can be influenced and altered by the TME. The model allows compartmentalization of the immune cells for ease of isolation and analysis while exposing them to the multicellular crosstalk from the TME.

The LumeNEXT platform has been successfully used to model endothelial vessels [20, 32, 33, 40] and the breast cancer microenvironment [25, 41–43]; therefore, we used this platform as the basis for the prostate ductal model. To restrict the variation in the system, we opted to use the BCaP cell lines to line the prostate duct. These cells, derived from a BPH cell xenograft treated over time with estrogen and testosterone, mimic the stages of cancer progression from non-tumorigenic cells (NT) through increases in tumor mass and eventually metastatic progression (M1) [24].

We chose to add PBMCs to the center of the lumen as it allowed the immune cells to be exposed to the multicellular crosstalk of the benign and metastatic microenvironments but provided easy access to remove the PBMC for analysis. The experimental design allowed a greater number of PBMC to be added to the model, which permitted analyses such as the qPCR array that would be difficult to achieve with smaller numbers of embedded cells. One limitation of this approach is that this organization does not exactly mimic that *in vivo* where immune cells would be located in the stromal space. Future studies could investigate the impact of the system geometry by studying metabolism or behavior of PBMCs that were cultured in the stromal space compared to the lumen. The flexibility of the platform is a major advantage as it can be adapted to maximize experimental efficiency or to more closely mimic the *in vivo* environment depending on the user’s needs.

PBMCs within the platform showed high viability. One advantage of the platform is ease of imaging, allowing the models to be monitored with two-photon autofluorescence imaging of the optical redox ratio (intensity of NAD(P)H/intensity of FAD), which provides non-invasive insight into the metabolic state of single cells over time [44]. Using this approach, we revealed that immune cells exposed to the metastatic versus normal microenvironments showed several differences. The subpopulation analysis suggested that PBMCs in metastatic microenvironments had two very distinct metabolic populations, while PBMCs in normal microenvironments had two very similar metabolic populations at day 4 (Fig. 5C). This could be suggestive of different patterns of immune cell differentiation or activation in metastatic compared to normal microenvironments. The increase in redox ratio on day 4 in the immune cells exposed to the TME compared to immune cells in normal lumens is consistent with an increase in glycolysis for immune cells in the TME. Interestingly, increases in glycolytic metabolism and optical redox ratio are observed in activated T cells as well as in macrophages with the TAM phenotype [38, 39, 45, 46]. This non-invasive imaging allows multiplexed readouts of immune cell phenotyping.

The immune cells showed different patterns of gene expression depending on whether they were cultured in the benign or metastatic environment. Exposure to the metastatic microenvironment resulted in increases in gene expression for IL-1α and GM-CSF with concomitant increases in protein expression. IL-1α is expressed by a number of cells in the TME with hematopoietic sources including activated T cells and macrophages [47]. A pro-inflammatory cytokine, IL-1α, has been implicated in angiogenesis and invasiveness through evidence in mouse models [48] and is increased in the serum of prostate cancer patients when compared to normal donors [49]. However, the role of GM-CSF is less clear in prostate cancer, with some evidence suggesting that it can stimulate the immune response against the tumor; however, there is a larger body of evidence showing that it can stimulate tumor progression [50]. Furthermore, GM-CSF can drive the generation of immunosuppressive MDSC cells from CD33^+^ cells [51]. We also determined that soluble PD-1 levels were elevated in the protein secretion measurements from the tumor models, although there was not a significant increase in PD-1 gene expression across all five donors. Five mRNA splice variants for PD-1 have been identified in PBMC [52]. The full-length variant contains five exons and is homologous with membrane-bound PD-1. The other variants are alternatively spliced and lack certain exons. sPD-1 is homologous with a variant that lacks exons 2 and 3 [53]. While overall PD-1 gene expression did not change, it is possible that the relative amount of each transcript was altered, and the sPD-1 variant was upregulated. This could be interesting to investigate in future work using primers specific to each PD-1 splice variant. Alternatively, it is possible that the PD-1 primers used in the PCR array only recognize the full-length variant and were unable to detect the Δexon2/3 variant that codes sPD-1, resulting in the discrepancy between PD-1 gene expression and soluble PD-1 levels. While the function of soluble PD-1 is still relatively unknown, there have been reports that soluble PD-1 can inhibit T-cell function through binding to PD-L1 on dendritic cells and reverse signaling from the APC to the T cell [54]. In addition, gene expression for STAT1 and STAT3 was also significantly downregulated. Evidence from STAT1 knockout mice models suggests that loss of STAT1 signaling results in enhanced tumor growth and increased number of exhausted T effector cells and TAMs [55]. We also observed downregulation of TRAIL gene expression, which is a death receptor expressed on the surface of T and NK cells that induces apoptosis in tumor cells when ligated to death receptors [56] and knocking out the TRAIL receptor results in increased susceptibility to tumor initiation and metastases [57]. Taken together, these results suggest that exposure of immune cells to the TME in our model system resulted in metabolic changes as well as gene and protein expression changes that can dampen the immune response to the tumor.

For this study, we chose to use PBMC from normal donors as we wanted to investigate the development of tumor-associated phenotypes in cells with no previous exposure to the TME. However, there are differences between the immune cells found in normal blood and those in the TME. In the TME, monocytes will quickly differentiate into TAMs, which span a heterogenous spectrum of phenotypes between suppressive and tumoricidal [58, 59]. Additional immunosuppressive cells are often enriched in the TME including regulatory T cells [37] and myeloid-derived suppressor cells [60]. While we saw changes in gene expression patterns and secreted factors that suggested changes in the immune cell phenotypes, we were not able to determine how closely the immune cells from the metastatic model correlate with immune cell populations in the tumor. In this study, due to limitations of flow cytometry, it was challenging to further phenotype the immune populations. In the future, advances in phenotyping small numbers of cells would provide additional information that would be helpful to further interpret the data presented here. Additional phenotyping of fibroblast populations would also be of interest to determine how these populations change over time and how these phenotypes would influence immune cell phenotype and function. Immune cells isolated from areas of normal tissue from prostatectomy samples would represent an interesting future source of immune cells for use in phenotyping studies in this system, which may have more biological relevance than PBMCs.

This report has focused on model development, demonstrating several advantages for multiplexed molecular analyses of immune cells exposed to the prostate TME. Each component of the model can be manipulated to determine the relative role of cell types, for example removing fibroblasts or depleting CD14^+^ cells. The potential also exists to make patient-specific models using matched epithelial and fibroblasts isolated from prostate tissue, and while we have placed immune cells into the lumen for ease of analysis in this model, embedding them in the matrix surrounding the prostate duct could create an organotypic model that more accurately recapitulates the TME architecture for different study goals. While most organotypic models aim to mimic the *in vivo* environment more closely, here we have demonstrated that designing models for functional analysis can also be a sound strategy to allow investigation of complex systems such as human tumor immunology.

## Supplementary Material

FigureS1_zyaa020

Supplemental_figure_2_zyaa020
