# Supplementary figures and images for: A bioengineered organotypic prostate model for the study of tumor microenvironment-induced immune cell activation
Source: Integr Biol (Camb). 2020 Oct 9;12(10):250–62. doi: 10.1093/intbio/zyaa020 (PMC7569006; doi:10.1093/intbio/zyaa020)

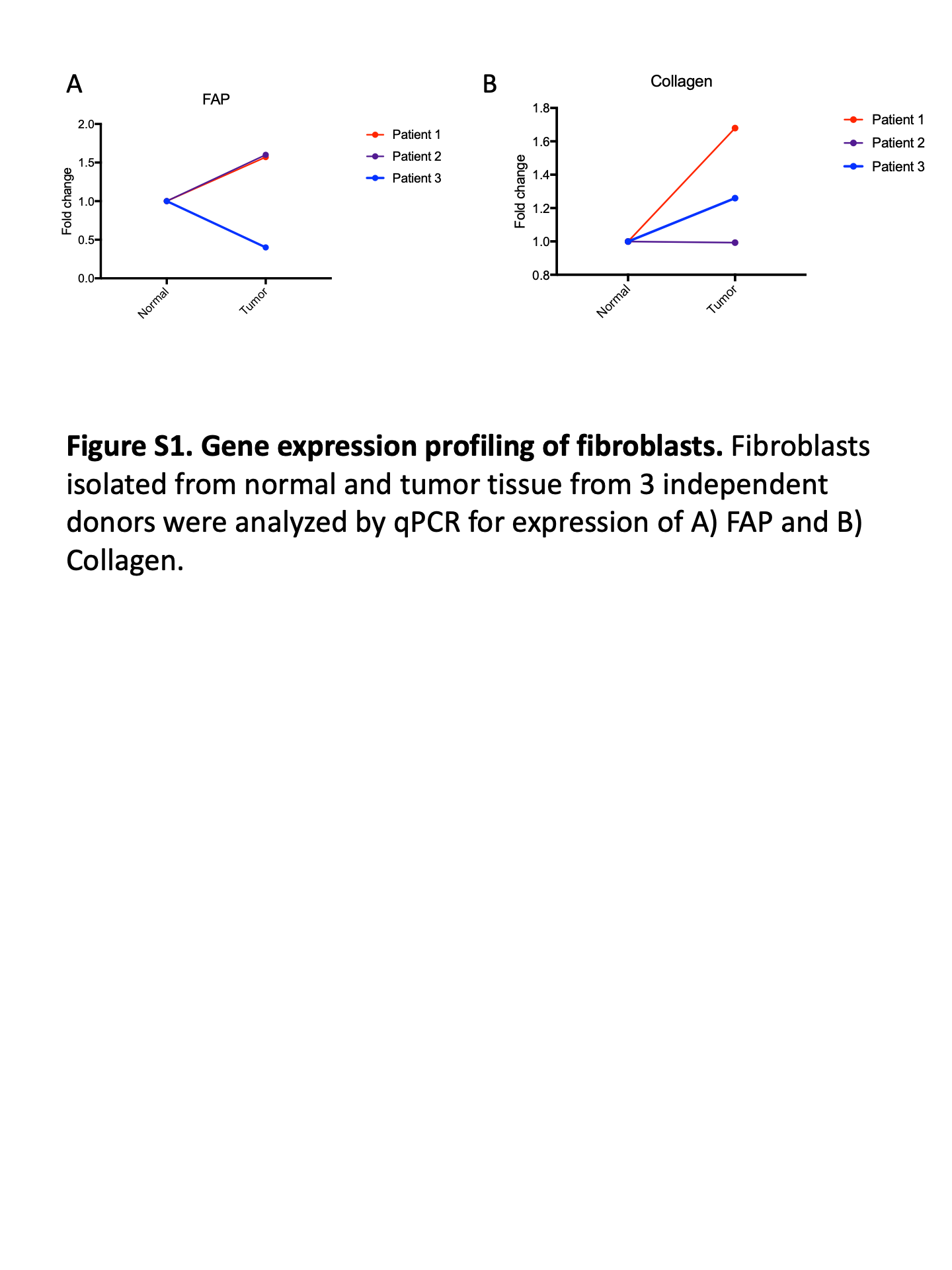

Supplement: FigureS1_zyaa020 [file figures1_zyaa020.png]

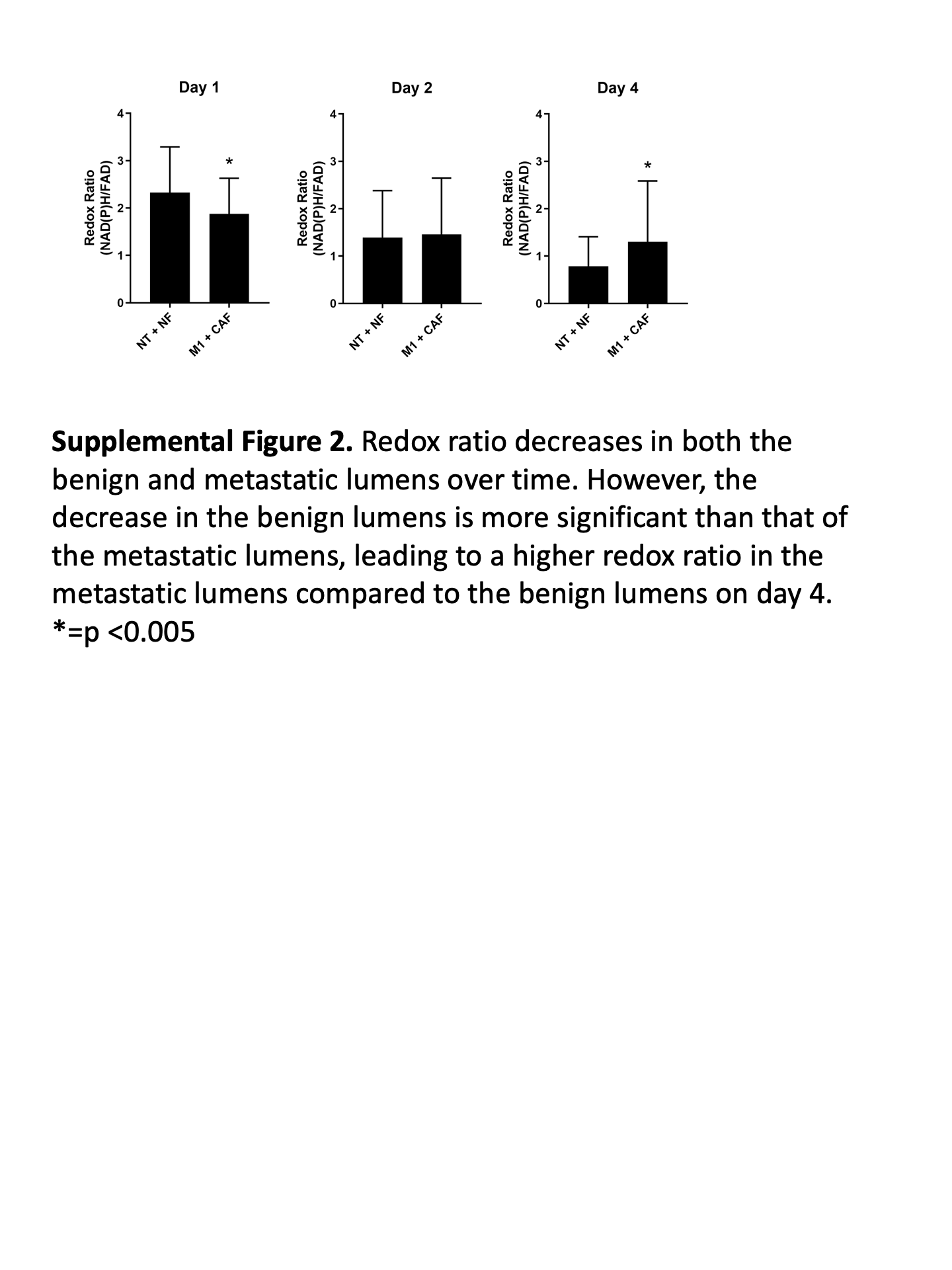

Supplement: Supplemental_figure_2_zyaa020 [file supplemental_figure_2_zyaa020.png]
